# Progress on Structured Biosensors for Monitoring Aflatoxin B1 From Biofilms: A Review

**DOI:** 10.3389/fmicb.2020.00408

**Published:** 2020-03-27

**Authors:** Qi Wang, Qingli Yang, Wei Wu

**Affiliations:** College of Food Science and Engineering, Qingdao Agricultural University, Qingdao, China

**Keywords:** aflatoxin B1, detection, aptasensors, aptamers, immunosensor, biosensors

## Abstract

Aspergillus exists commonly in many crops and any process of crop growth, harvest, storage, and processing can be polluted by this fungus. Once it forms a biofilm, Aspergillus can produce many toxins, such as aflatoxin B1 (AFB1), ochratoxin, zearalenone, fumonisin, and patulin. Among these toxins, AFB1 possesses the highest toxicity and is labeled as a group I carcinogen in humans and animals. Consequently, the proper control of AFB1 produced from biofilms in food and feed has long been recognized. Moreover, many biosensors have been applied to monitor AFB1 in biofilms in food. Additionally, in recent years, novel molecular recognition elements and transducer elements have been introduced for the detection of AFB1. This review presents an outline of recent progress made in the development of biosensors capable of determining AFB1 in biofilms, such as aptasensors, immunosensors, and molecularly imprinted polymer (MIP) biosensors. In addition, the current feasibility, shortcomings, and future challenges of AFB1 determination and analysis are addressed.

## Introduction

A biofilm is an extracellular matrix secreted by biological flora and easily adheres to biological or non-biological surfaces (Srey et al., 2013; Xu et al., 2019). Biofilm formation represents a self-protection mechanism of bacteria and fungi. Moreover, the biofilms formed by Aspergillus intensively produce many toxins in food. It is commonly accepted that the infection and proliferation of biofilm mycotoxins may occur in any field, harvest, and storage process (Siegel and Babuscio, 2011). Mycotoxins are low-molecular-weight natural secondary metabolites produced by certain fungi (Krittayavathananon and Sawangphruk, 2017). AFB1 is the most toxic among all mycotoxins, posing teratogenic, carcinogenic, and mutagenic risks to humans, and has been labeled as a group I carcinogen in humans by the International Agency for Research on Cancer (IARC) (IARC, 2002; Abnous et al., 2017a). In addition, AFB1 commonly exists in many crops such as grain, peanut, corn, and feed. AFB1 production and pollution can occur during all processes along the food chain. Because AFB1 results in significant health and economic problems in many countries, AFB1 contamination is one of the most serious problems threatening food safety (Uludag et al., 2016; Xue et al., 2019). Therefore, it is necessary to exploit novel, low-cost, and fast on-site detection technology as well as miniaturized instruments for real-time monitoring of AFB1 and prevention of AFB1 contamination.

Traditionally, aflatoxin B1 (AFB1) detection is performed by thin-layer chromatography (TLC) (Var et al., 2007; Casoni et al., 2017), enzyme-linked immunosorbent assay (ELISA) (Lee et al., 2004), mass spectrometry (MS), gas chromatography (GC), liquid chromatography (LC) (Jin and Choi, 2007; Fan et al., 2015), and high-performance LC (HPLC) (Ghali et al., 2009). These detection assays benefit from having a high sensitivity and mature technology. However, these methods require high-cost instruments and equipment, long test times, and skilled lab researchers for the detection process. These shortcomings have limited the development of these methods for mycotoxin detection to a certain extent. Moreover, biological sensors are a new, emerging technology for the determination of mycotoxins.

**FIGURE 1:**
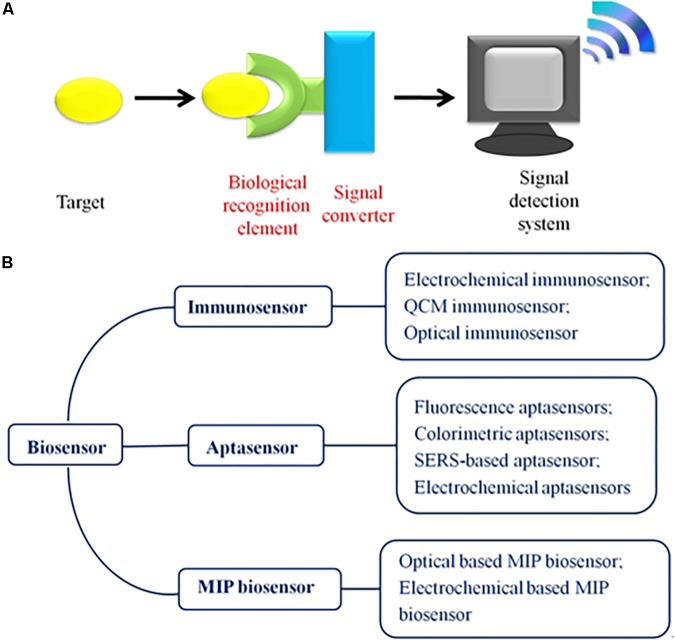
**(A)** Schematic illustration of the biosensor, including the following three parts: the bio-recognition element, the signal converter, and the signal measurement system. **(B)** Outline of the biosensors used for monitoring AFB1. According to the bio-recognition element, the biosensor is divided into aptasensors, immunosensors, and MIP biosensors in this review.

A biosensor is a kind of detection method used to convert biological signals into electrical signals. This detection method offers an excellent performance, as it is easy-to-use, inexpensive, very specific, and highly sensitive. Generally, a biosensor includes three main parts: a bio-recognition component, a signal converter, and a signal measurement system (Figure 1A). The bio-recognition element is the core part of a biosensor, and common bio-recognition elements include aptamers (Alizadeh et al., 2018; Danesh et al., 2018), antibodies (Eivazzadeh et al., 2017), molecularly imprinted polymers (MIPs; Ton et al., 2015), and enzymes (Ricard and Buc, 2005). These bio-recognition elements possess a high selectivity and specificity for specific target substances, and only in this way can biological sensors achieve better selectivity. In addition, the signal converter is closely connected to the biological recognition component. First, the target molecules are captured by the biological recognition component. Then, the signal converter converts the biological signals into physical signals, including electrical signals, fluorescence signals, magnetic signals, and so on. Finally, these signals are detected by the detection system. Sometimes, the signal generated by the signal converter will be amplified by the signal amplifier before reaching the detection system. To date, biosensors have been used in many fields, including pathogen (Khansili et al., 2018) toxin and pesticide residue (Shang et al., 2011) detection in food, bio-marker detection for medical diagnostics, detection in water (Han et al., 2013), and detection in the atmosphere. In recent years, the combination of biosensors and nanomaterials [quantum dots (QDs), carbon nanomaterials, noble metal nanoparticles, and magnetic nanoparticles] has attracted the attention of researchers (Farka et al., 2017; Xue et al., 2019).

Based on the differences between the bio-recognition elements, biosensors have been classified as aptasensors, immunosensors, and MIP-based biosensors (Figure 1B). Herein, this review focuses on biosensors developed for AFB1 detection in the past 5 years. We aim to evaluate the superiority and limitations of the reported biosensors in overcoming the challenges and drawbacks of their applications.

## Aptasensors for Aflatoxin B1 From Biofilm

An aptasensor is a biosensor that uses aptamers as the recognition element. Aptamers are short, single-stranded oligonucleotide sequences (DNA, RNA, or nucleic acid analogs) selected from a nucleic acid molecular library using the *in vitro* systematic evolution of ligands by exponential enrichment (SELEX) method (Stoltenburg et al., 2007; Abnous et al., 2017b; Alizadeh et al., 2018). Owing to their dimensional folded configurations, aptamers possess a high specificity and affinity for specific targets, including mycotoxins, pathogens, metal ions, pesticides, and cells (Meng et al., 2015; Danesh et al., 2018; Liu et al., 2019; Wu et al., 2019c, d; Yu S.H. et al., 2019). In contrast to antibodies, aptamers possess a superior sensitivity and stronger stability toward various pH values, temperatures, and ions, can be easily synthesized *in vitro* and modified, and are inexpensive (Rothlisberger and Hollenstein, 2018; Wu et al., 2019c). Therefore, the latent recognition ability of aptasensors for use as biosensors is better than that of immunosensors. So far, aptasensors have attracted a great deal of attention and have created new approaches for the sensitive and selective detection of toxins (Yugender Goud et al., 2017; Qian et al., 2018; Ma et al., 2019; Wang J. et al., 2019; Wu et al., 2019b). Additionally, various aptasensors have been utilized for AFB1 detection, including chemiluminescent aptasensors, fluorescent aptasensors, surface-enhanced Raman scattering (SERS)-based aptasensors, colorimetric aptasensors, and electrochemical aptasensors. Herein, we classified and comprehensively evaluated the reported aptasensors for monitoring AFB1. In addition, aptamer sequence, LOD and linear range of various aptasensors were listed in Table 1.

**TABLE 1:** Selected examples of aptasensors for detection of AFB1.

**Detection methods**	**Aptamer sequence (5′–3′)**	**LOD**	**Linear range**	**References**
Fluorescence	GTT GGG CAC GTG TTG TCT CTC TGT GTC TCG TGC CCT TCG CTA GGCCC	20 pg/mL	0.05–100 ng/mL	Lu et al., 2019
	GT TGG GCA CGT GTT GTC TCT CTG TGT CTC GTG CCC TTC GCT AGG CCC ACA	0.3 pg/mL	5–1000 pg/mL	Nasirian et al., 2017
	AAA AAA AAG TTG GGC ACG TGT TGT CTC TCT GTG TCT CGT GCC CTT CGC TAG GCC CAC AC	70 pg/mL	0.1–0.8 ng/mL	Li Z. et al., 2019
	ATA TCT TTT CCT ACT CAT CTT TGA ATA ACT ACC GGG CAT TAC TTT CTG GCC TCC CTG CCT CCT AAA TCA CCA ATT AAT TCG CGG CCC CCC G	4 ng/mL	0.002–0.2 μg/mL	Kumar et al., 2018
Colorimetry	GTT GGG CAC GTG TTG TCT CTC TGT GTC TCG TGC CCT TCG CTA GGC CC	1 pM	–	Wu et al., 2019a
SERS	GTTGG GCA CGT GTT GTC TCT CTG TGT CTC GTG CCC TTC GCT AGG CCC	3.6 pg/mL	0.01–100 ng/mL	Chen et al., 2018
	GTTGG GCA CGT GTT GTC TCT CTG TGT CTC GTG CCC TTC GCT AGG CCC	0.54 pg/mL	0.001–10 ng/mL	Yang et al., 2017
SPR	TGG GCA CGT GTT GTC TCT CTG TGT CTC GTG CCC T	0.4 nM	0.4–200 nM	Sun et al., 2017
	GTT GGG CAC GTG TTG TCT CTC TGT GTC TCG TGC CCT TCG CTA GGC CCA CA	0.19 ng/mL	1.5–50 ng/mL	Wu et al., 2018
Electrochemistry	GTT GGG CAC GTG TTG TCT CTC TGT GTC TCG TGC CCT TCG CTA GGC CCA CA	0.01 fg/mL	0.1 fg/mL-0.1 μg/mL	Peng et al., 2018
	GTT GGG CAC GTG TTG TCT CTC TGT GTC TCG TGC CCT TCG CTA GGC CCA CA	86 fg/mL	0.1–10 ng/mL	Selvolini et al., 2019
	GTT GGG CAC GTG TTG TCT CTC TGT GTC TCG TGC CCT TCG CTA GGC CCA CA	0.13 ng/mL	1-20 ng/mL	Mo et al., 2018

### Fluorescent Aptasensors

Fluorescence spectrometry is a practical method for the sensitive determination of samples with low quantitative amounts (Gao et al., 2018; Yang Y. et al., 2018). In recent years, coupling with fluorescent nanomaterials, such as carbon dots (CDs), fluorescence dyes, up-conversion nanoparticles (UCNPs), QDs, metal nanoparticles [e.g., gold nanoparticles (AuNPs) and silver nanoparticles (AgNPs)], has become a trend in fluorescent aptasensors (Huang et al., 2018; Yang C.Y. et al., 2018; Li Z. et al., 2019; Wang Y.J. et al., 2019; Zhang M.M. et al., 2019). The most commonly used strategy of fluorescent aptasensors is the *signal-on* method, except for some cases that typically apply the theory of fluorescence resonance energy transfer (FRET). On the other hand, *signal-off* fluorescent aptasensors usually cannot eliminate the potential experimental uncertainties and false positives caused by the fluorescence source itself. According to FRET, fluorophores are used as fluorescence donors, and quenchers are used as fluorescence acceptors. First, fluorescence is blocked by the quencher, forming a detection platform in the fluorescence *signal-off* state. When the target analytes are added, the fluorophore-modified aptamer would release from the quencher surface due to the binding affinity of the aptamer and target being stronger than that of the aptamer and quencher, and the aptamer would subsequently combine with the targets and yield a significant fluorescence intensity. In addition, metal nanoparticles, humic acid (HA) (Guo M. et al., 2019), graphene oxide (GO) (Wang et al., 2020), and a quenching group have frequently been used as fluorescence quenchers.

**FIGURE 2:**
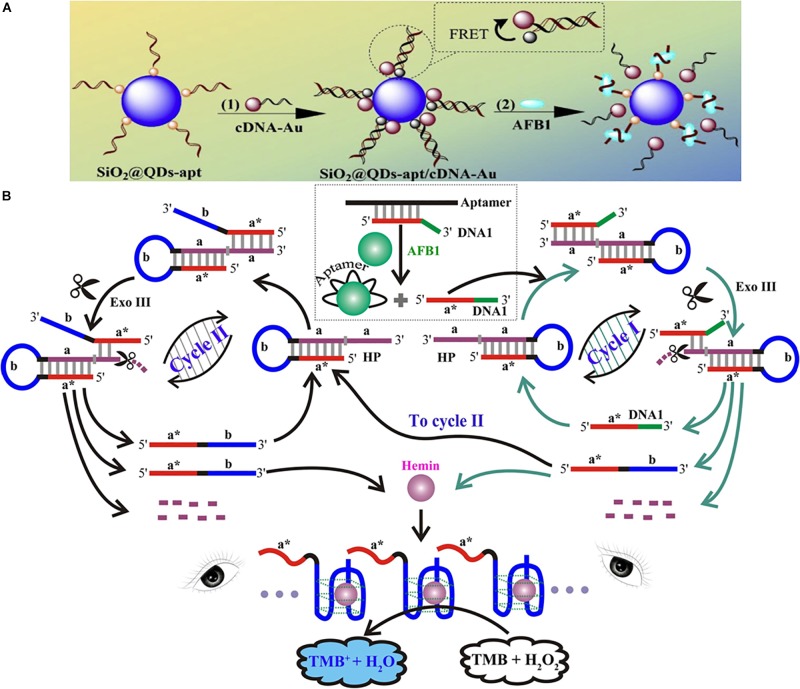
Different assays of optical aptasensors for AFB1 detection. **(A)** Schematic illustration of ternary QDs based fluorescent assay. **(B)** Schematic illustration of G-quadruplex DNAzyme-based colorimetric assay. Modified from Lu et al. (2019) and Wu et al. (2019a).

Metal nanoparticles (AuNPs or AgNPs) are usually used as fluorescence acceptors due to their high extinction coefficient and powerful quenching ability (Farka et al., 2017; Xue et al., 2019). Recently, Lu et al. (2019) employed fluorescence switch-on aptasensors for the determination of AFB1 based on the FRET mechanism between CdZnTe QDs and AuNPs (Figure 2A). Therein, highly fluorescent ternary CdZnTe QDs were successfully prepared. After incubation of the CdZnTe QDs-aptamer and AuNPs-cDNA, the fluorescence of the QDs was blocked by the AuNPs because of the DNA hybridization that occurred between the aptamer and cDNA. When the target was added, the aptamer preferred to combine with AFB1 because the aptamer had a higher affinity for AFB1 than for the target, resulting in the fluorescence recovery of the CdZnTe QDs and detachment of cDNA-AuNPs. In addition, the LOD of this work was shown to be 50 pg/mL. Different metal nanoparticles have been used for AFB1 detection. For instance, Nasirian et al. (2017) established a FRET platform for the ultrasensitive determination of AFB1 that depended on the adsorption and fluorescence quenching ability of AgNPs-cDNA to a polymer dots-aptamer. Interestingly, the LOD of that work was shown to be 0.3 pg/mL.

A material with a strong affinity for single-stranded DNA (ssDNA), such as HA and GO, can also be employed to construct FRET platforms. The former, HA, possesses abundant quinoid units, aromatic rings, and sugar moieties. The latter, GO, possesses a large amount of oxygen-containing functional groups on its surface. Owing to π–π stacking, the aptamer is adsorbed on the GO surfaces. Li et al. developed a novel fluorescence aptasensor using CD-modified aptamers as the capture probes and HA as the quencher for AFB1 detection, and the LOD was 70 pg/mL (Guo M. et al., 2019). Compared with conventional metal QDs, CDs have the following benefits: they are easy to synthesize, environmentally friendly, green, non-toxic, derived from abundant sources, inexpensive, biodegradable, and so on. In addition, Poda’s group assembled a GO-FRET platform by utilizing a QD-aptamer and GO for AFB1 detection, and the LOD was 0.004 μg/μL (Kumar et al., 2018).

A quencher group, such as black hole quencher 1 (BHQ1) and tetramethyl-6-carboxyrhodamine (TAMRA), has also been used in the quenching system. Generally, fluorophores, such as FAM (carboxyfluorescein), are modified with an aptamer, and the quencher group is modified with cDNA. In the absence of a target, the fluorescence is blocked due to the base complementary condition of the aptamer and cDNA. When a target is added, fluorescence is recovered and cDNA is detached. Taking advantage of these properties, Xia et al. (2019) constructed a dual-terminal proximity structure detection platform by utilizing the FAM-aptamer and anti-aptamer-labeled BHQ1. In this aptasensor, AFB1 competitively combines with the aptamers, resulting in the destruction of the dual-terminal proximity structure. Additionally, the aptasensor can be used to implement ultrafast determination in one minute. Therefore, this work produced an immensely successful aptasensor for the rapid determination of AFB1.

A label-free method can obtain direct evidence by detecting analytes without a label. Thus, label-free biosensors are one of the most widely used detection methods. In fact, most of the reported aptasensors were designed using the label-free approach. Jia et al. (2019) reported a new label-free fluorescent aptasensor for monitoring AFB1 in food samples by employing aggregation-induced emission (AIE) molecules and GO. Jia et al. considered the possibility of traditional fluorescence dye self-quenching in an aggregated state. Therefore, quaternized tetraphenylethene salt (TPE-Z), a kind of AIE molecule, was used as the label-free fluorescence dye. In this work, the LOD was 0.25 ng/mL.

### Colorimetric Aptasensors

Colorimetry is a convenient method for *in situ* detection because the detection results can be observed by the naked eye without using an instrument. When the targets are added, the colorimetric aptasensors can convert the target signal into a color change. To improve the sensitivity, the signal amplification strategy has been employed in increasingly more widespread applications in colorimetric biosensors for detecting low analyte concentrations (Taghdisi et al., 2018; Li C. et al., 2019). In the colorimetric aptasensor system, noble metal nanoparticles are usually applied as signal indicators due to their ability to change color when changing from a dispersion state to an aggregation state (Danesh et al., 2018; Zhang et al., 2018). Enzyme catalysis is another common method used to change the color.

A colorimetric biosensor based on the nuclease-assisted signal amplification strategy was fabricated for the naked-eye detection of AFB1 (Figure 2B) (Wu et al., 2019a). In this work, the domain **a^∗^** of DNA1 and the AFB1 aptamer were hybridized together in the absence of AFB1, preventing the combination of DNA1 and the hairpin DNA probe (HP). The HP included the stem region (domains **a,** which was the recognition unit, and **a^∗^,** which was complementary to **a**), and **a** surrounded the G-rich sequence lying in the loop domain (domain **b**). The aptamer preferred to combine with AFB1 in the presence of this toxin, releasing DNA1. Then, DNA1 and HP combined based on the principle of base complementarity, forming the blunt or recessed 3′ termini of the HP. At this time, Exo III could cleave duplex DNA, liberating DNA 1 to re-enter the above-mentioned cycle (cycle I). Moreover, a new DNA fragment (domains a^∗^ and b of DNA 2) could participate in the next cycle (cycle II), in which HP catalyzed the cleavage of mononucleotides to form DNA 2 by Exo III. At the end of the cleavage reaction of cycles I and II, the G-rich oligomer of the exponentially growing DNA 2 and co-factor hemin could assemble into active DNAzyme. Then, the G-quadruplex DNAzyme could catalyze the oxidation reaction of H_2_O_2_ and TMB, and the color of the system would change from colorless to blue. Note that this work represented a brilliantly designed colorimetric aptasensor based on the signal amplification principle. In addition, the new DNA fragment (DNA 2) played a crucial role in the recycling process.

Another example employed enzyme-free amplified colorimetric aptasensors based on AuNPs for AFB1 determination (Chen et al., 2016). The signal amplification system was assembled by three biotinylated hairpin DNA probes (H1, H2, and H3). In the absence of AFB1, the aptamer-based T-DNA combined with DNA (B). However, the aptamer-AFB1 complex would activate the signal amplification device when AFB1 was added. T-DNA subsequently opened the hairpin structure of H1, H2, and H3, further forming the T–H1–H2–H3 complex. However, T-DNA would dissociate from the T–H1–H2–H3 complex, continuing to open the left hairpins. In this aptasensor, streptavidin functionalized AuNPs were used as colorimetric indicators. Then, biotinylated H1–H2–H3 complexes would combine with AuNPs via streptavidin–biotin interaction, forming a crosslinked network of AuNPs. The ultimate red-to-blue color variation can be distinguished by the naked eye.

### SERS Aptasensors

Surface enhanced Raman scattering (SERS) is an extension of the spectroscopic method developed on the basis of Raman spectra and metal nanoparticles (AuNPs or AgNPs) (Lee et al., 2019; Yu B.R. et al., 2019). Because metal nanoparticles possess an excellent signal amplification effect, the sensitivity level of SERS can be equivalent to that of fluorescence (Ding et al., 2017). The SERS aptasensors not only provide a label-free approach, which simplifies the steps and saves costs, but also possess an ultrahigh sensitivity, even down to the single-molecule level.

**FIGURE 3:**
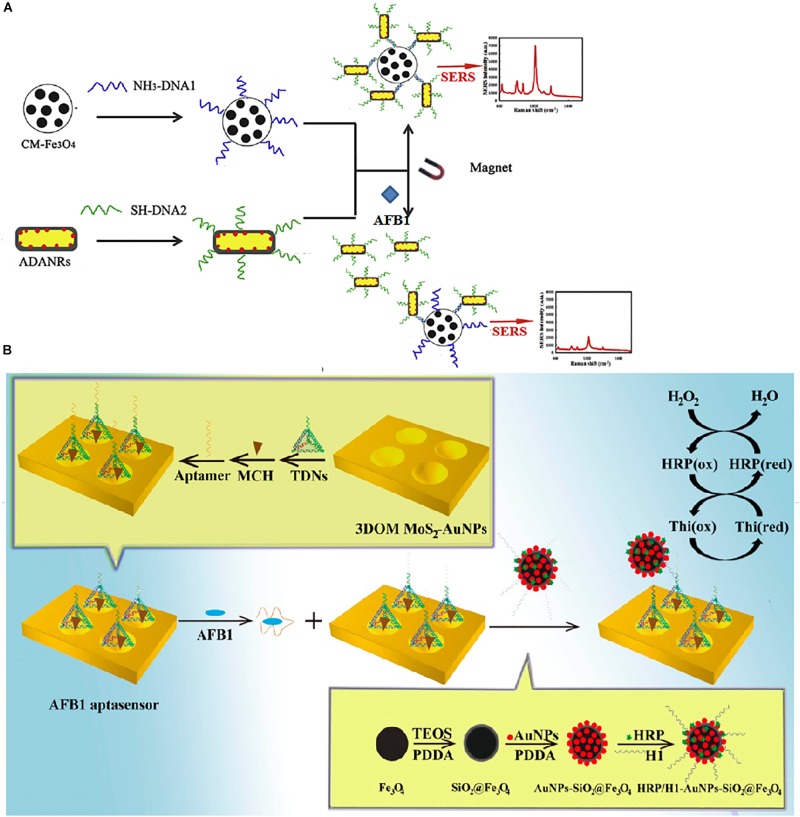
Different aptasensors for AFB1 determination. **(A)** Schematic illustration of AuNPs-based SERS assay. **(B)** Schematic illustration of HPR based electrochemical assay. Modified from Chen et al. (2018) and Peng et al. (2018).

Due to the stability and sensitivity of the SERS signal, core-shell nanoparticles have been widely employed in SERS sensors. One SERS aptasensor was assembled by aptamer-CS-Fe_3_O_4_ and AFB1-complementary aptamer-AuNR@DNTB@Ag nanorods (ADANRs) (Figure 3A) (Chen et al., 2018). ADANRs are SERS reporter nanoprobes with a core-shell structure and produce a very sensitive SERS signal. When AFB1 was added, this compound preferred to combine with the aptamer, leading to the dissociation of the aptamer-CS-Fe_3_O_4_ and cDNA-ADANRs. In addition, the SERS signal at 1331 cm^–1^ decreased, and the ADANRs were released. Using this SERS aptasensor, AFB1 was monitored at concentrations as low as 0.0036 ng/mL. Chen et al. also employed novel core-shell nanoparticles [gold nanotriangles (GNTs)-DTNB@Ag-DTNB nanotriangles] as reporters for AFB1 determination (Yang et al., 2017). The Raman characteristic peak of AFB1 is 1331 cm^–1^. The LOD was 0.54 pg mL^–1^, and the linear range was from 0.001 to 10 ng/mL.

### Electrochemical Aptasensors

Due to their outstanding advantages, which include fast detection, easy operation, and low cost, electrochemical biosensors have been widely utilized in medical, food, and environmental fields (Wu et al., 2018). There is growing interest in employing electrochemical aptasensors that combine aptamers with electrochemical analysis technology for analyte detection.

A novel AFB1 electrochemical aptasensor based on a stereoscopic regulated macroporous MoS_2_-AuNP film (SRM MoS_2_-AuNPs) was constructed and used as a horseradish peroxidase (HRP)-modified electrode (Figure 3B) (Peng et al., 2018). In this work, the AFB1 aptamer could hybridize tetrahedral DNA nanostructures (TDNs), which were immobilized on the modified electrode. In the presence of AFB1, the aptamer preferentially combined with the toxin, forming base vacant TDNs. Thus, the TDNs could bind with the complex of the helper strand (H1)/HRP-modified nanospheres due to the base complementarity of H1 and the TDNs. HRP could catalytically reduce H_2_O_2_ to produce one electron. There was a linear relationship between the current response and the AFB1 concentration. Moreover, the detection limit of this brilliantly designed aptasensor was 0.01 fg/mL.

Selvolini et al. (2019) reported novel electrochemical aptasensors based on competitive approaches using AFB1 and bovine serum albumin (AFB1–BSA). The AFB1–BSA complex was coupled on the surface of graphite screen-printed electrodes. In addition, free and immobilized AFB1 molecules competed to combine with the aptamer, and the streptavidin-alkaline phosphatase enzyme conjugate monitored this process. The LOD of AFB1 was 0.086 ng/mL.

A porous anodized alumina (PAA) membrane can be used to construct nanostructured arrays. Nanochannel sensors are sensitive to electric charge change. Mo et al. (2018) developed novel sandwich structures of electrochemical aptasensors using a PAA membrane, aptamer, and GO to monitor AFB1. In the presence of AFB1, GO detached from the nanochannel surfaces, causing a decrease in the steric effect and charge density. Therefore, the current response increased as Fe(CN)_6_^3–^ passed through the nanochannels.

## Immunosensors

Immunosensors are the most mature monitoring method for rapid detection and combine immunoassays and biosensor technology. Immunosensors can convert the recognition of an antibody toward a specific antigen into a detection signal. Normally, the antibody is an immunoglobulin secreted by B lymphocytes in the immune system when the body is infected by antigens. Although other recognition elements have been applied in the detection field, classic antibodies, as the most popular recognition components, still dominate most markets in the field of study and commercial affinity assays.

### Electrochemical Immunosensors

In electrochemical biosensors, the recognition element is mostly immobilized on the surface of electrodes. Therefore, electrochemical immunosensors can convert the recognition of an antibody toward a specific target into a detectable electrochemical signal (current, resistance, and potential). In this part of the review, electrochemical techniques, including electrochemical impedance spectroscopy (EIS), cyclic voltammetry (CV), and photoelectrochemical (PEC) methods, are discussed.

We found that EIS and CV were frequently used together in these studies. EIS measures the ratio of voltage to current at a specific frequency. In this way, it is easier to analyze the data. EIS is a detection method of the frequency domain, and this technique can monitor a wide frequency range. CV is one of the most popular electrochemical techniques and measures the current response. In addition, the sensitivity of the biosensor is determined by the sensitivity of the electrode to a change in the material. To improve the sensitivity of electrodes, nanomaterials—such as AuNPs, QDs, magnetic beads, and carbon nanomaterials—are increasingly applied to electrochemical immunoassays. Among these nanomaterials, AuNPs are commonly used as signal amplification labels due to their excellent catalytic, electrical, optical, and chemical properties. Bhardwaj et al. (2019) described an approach in which graphene QDs (GQDs) and AuNP-based electrochemical immunosensors were used to detect AFB1. Here, antibodies against AFB1 were immobilized on the surface of an ITO glass electrode coated with the GQD-AuNP composite. CV and EIS techniques were both used to evaluate the electrochemical response of this immunosensor. The edge effects of the GQDs dramatically increased the rate of heterogeneous electron transport of the composite GQDs-AuNPs. Moreover, the electrocatalytic activity of the AuNPs improved the electronic properties of the composite GQDs-AuNPs. In this study, there was a linear relationship between the concentration of AFB1 and the current signal. In addition, the linear range was 0.1–3.0 ng/mL. Similarly, Li et al. (2017) constructed a label-free impedimetric immunosensor based on Au three-dimensional nanotube ensembles (3DTNEEs) and the AFB1 antibody. The AFB1 antibodies were immobilized on the 3DTNEEs using a staphylococcus protein A layer. In this study, the particular tube-like structure and the high surface areas of the 3DTNEEs effectively improved the sensitivity of the immunosensor. The LOD of AFB1 was 1 pg/mL. In another example, Costa’s group reported an impedimetric immunosensor based on carbon nanotubes and an Au electrode for monitoring AFB1 (Costa et al., 2017). In this immunosensor, the carbon nanotubes exhibited an exceptional surface/volume ratio and excellent electrical properties.

PEC can not only translate chemical energy produced by light into electrical energy but also provide high sensitivity and a low background signal. In addition, illumination electrodes play a crucial role in PEC biosensors. Zn_3_(OH)_2_V_2_O_7_⋅2H_2_O, a photoelectrochemically active material, can produce a photocurrent under UV light due to its wide band gap, but this characteristic is very weak for visible light absorption. Lin et al. (2017) synthesized novel composites with doped transition metal ions to improve the performance of Zn_3_(OH)_2_V_2_O_7_⋅2H_2_O. Moreover, dopamine-loaded liposomes were utilized to upgrade the photocurrent of Mn^2+^-doped composites. Considering the abovementioned advantages, Lin et al. developed a novel on-site PEC immunosensor based on signal amplification for monitoring AFB1. Importantly, the LOD of this PEC immunosensor was 0.3 pg/mL.

### Optical Immunosensors

Optical immunosensors used for AFB1 detection have been fabricated by fluorescence, SERS, surface plasmon resonance (SPR), and photoluminescence (PL) assays. Nanoparticles play vital roles in optical immunosensors. The core reasons might be due to the excellent optical properties of the nanomaterials and the sensitivity of the immunosensors. In this part, we compared various immunosensors based on optical monitoring assays. Optical immunosensors reported for monitoring AFB1 are reviewed in Table 2.

**TABLE 2:** Selected examples of optical immunosensors for detection of AFB1.

**Optical strategies**	**Nanometerials**	**LOD**	**Linear range**	**References**
Fluorescence	Magnetic fluorescent beads	27 ± 3 pg/mL	5-150 pg/mL	Guo M. et al., 2019
	CdTe/CdS/ZnS quantum dot	0.01 ng/mL	0.08–1.25 ng/mL	Zhang M.M. et al., 2019
	–	0.21 ng/mL	1.0–1000 ng/mL	Shu et al., 2019
	Porous g-C3N4 nanosheets	2 pg/mL	0.01–0.5 ng/mL	Xie et al., 2019
SPR	AuNPs	0.003 nM	0.01–50 nM	Bhardwaj et al., 2020
	–	0.59 ng/mL	–	Wei et al., 2019
	–	2.51 ppb	–	Moon et al., 2018
SERS	AuNPs	0.06 g/kg	–	Li et al., 2018
	Silica-encapsulated hollow AuNPs	0.1 ng/mL	1–10^5^ ng/mL	Ko et al., 2015
	Gold nanobipyramids	0.5 μg/L	–	Lin et al., 2020
PL	Gold-coated porous silicon nanocomposite	2.5 pg/ml	0.01–10 ng/ml	Myndrul et al., 2017

#### Fluorescence Immunosensors

**FIGURE 4:**
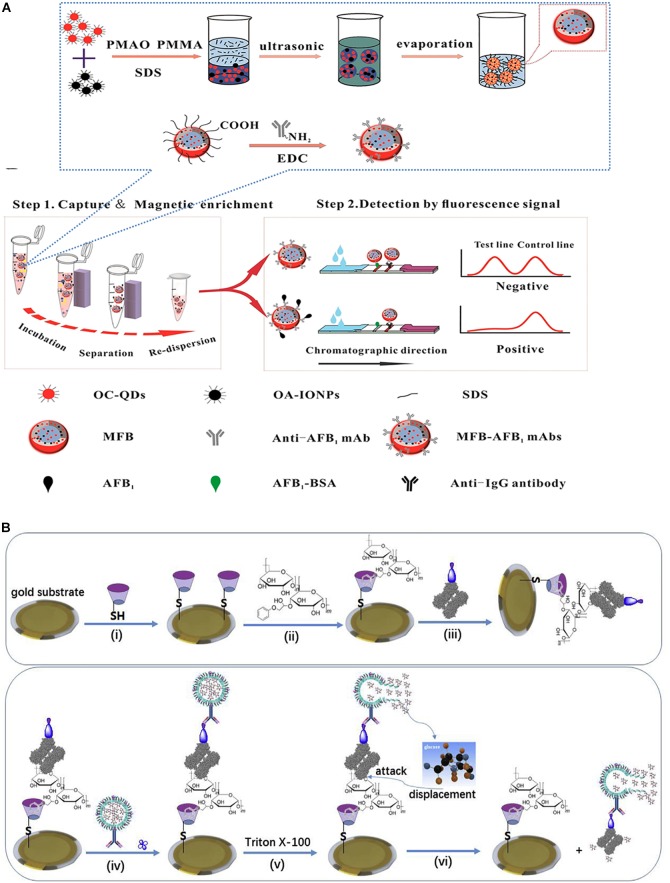
Different immunosensor for AFB1 detection. **(A)** Schematic illustration of magnetic QDs fluorescence-based assay. **(B)** Schematic illustration of QCM-based immunoassay. Modified from Tang et al. (2018) and Guo L. et al. (2019).

Guo L. et al. (2019) synthesized bi-functional magnetic fluorescent beads (MFBs) with a core/shell structure by using iron oxide nanoparticles and CdSe/ZnS QDs (Figure 4A). Anti-AFB1 antibody-labeled MFBs (MFB-mAbs) were used to fabricate MFB strips. MFBs were first reported as dual-functional probes for pre-concentrating the target and increasing the response of the competitive sensor. Under the optimal detection conditions, the detection of the biosensor reported in this work ranged from 5 to 150 pg/mL. In another example, Zhang F. et al. (2019) also employed CdTe/CdS/ZnS QDs for conjugation with an artificial antigen. Based on a one-step fluorescence immunoassay (FLISA), this immunosensor was developed for the accurate detection of AFB1.

#### SPR Immunosensors

Surface plasmon resonance is a practical and label-free optical sensing technology based on the differential refractive index changes of the molecular surface. In essence, SPR is generated from the resultant force of free charge oscillations and electromagnetic waves at the interface of the medium and metal (Zhao et al., 2019). Thus far, SPR biosensors have been employed in the fields of food (food allergens and mycotoxins), medicine (biomarkers and genes), and so on (Breveglieri et al., 2019; Jena et al., 2019; Wei et al., 2019; Zhou et al., 2019).

A new type of SPR immunosensor used for AFB1 determination using nanoparticles integrated into a gold chip was reported by Bhardwaj et al. (2020). Lipoic acid and cystamine could form a self-assembled monolayer (SAM) on the gold chip surface. AuNPs were immobilized on the SAM gold chip surface by an amine linkage. The SAM gold chip was carboxylated by EDC-NHS, combined with protein-A, and finally coupled with AFB1 antibodies. Using this approach, the linear range for monitoring AFB1 was 0.01–50 nM, with an LOD as low as 3 pM. In another example, Tao et al. established an SPR sensor chip based on a SAM for the simultaneous determination of AFB1, deoxynivalenol, zearalenone, and ochratoxin A in wheat and corn (Wei et al., 2019). The four antigens were immobilized on the SAM-SPR chip through a hydrazone linkage. Upon antibody addition, the binding index of the antibody and antigen was indicated by the SPR signal. Cross-reaction is a serious problem for many biosensors applied to simultaneously detect multiple targets. However, the low cross-reaction rate of antibodies demonstrates the high selectivity of the antibody to the antigen in this immunosensor. In addition, the ability to simultaneously detect multiple targets will become the development trend of biosensors.

#### SERS Immunosensors

Surface-enhanced Raman scattering assays have an advantage in that SERS signals do not exhibit self-quenching. In addition, Au/Ag nanoparticles are constantly used in SERS sensors. Li et al. (2018) explored an immunosensor based on SERS for the multiplexing determination of mycotoxins. In this study, AuNPs were applied as Raman labels and were combined with anti-mycotoxin antibodies by 5,5-dithiobis(succinimidyl-2-nitrobenzoate) (DSNB). The AuNP–DSNB–antibody complexes were used as SERS nanoprobes in which the Raman intensity of the DSNBs was greatly improved by AuNPs. The results showed a negative correlation between the concentration of AFB1 and the characteristic peak intensity in all spectra. In another example, a SERS immunosensor based on a sandwich approach was reported by Ko et al. (2015). Anti-AFB1-modified magnetic beads were used as the fixation material, and anti-AFB1-conjugated silica-encapsulated hollow AuNPs were employed to provide the SERS signal in this immunosensor; when AFB1 was added, the toxin combined with those two materials, forming a sandwich structure. The LOD of AFB1 was 0.1 ng/mL.

#### PL Immunosensors

Due to their portability and low cost, PL immunosensors are also very popular. Myndrul et al. (2017) applied a PL immunosensor based on macroporous silicon (PSi) blanketed by a thin gold (Au) layer to detect AFB1. The PSi/Au structures showed excellent PL properties. Here, protein A played a key role in coupling the PSi/Au structures and antibodies against AFB1. The linear range of the PSi/Au/protein-A/antibody-based immunosensors for AFB1 detection was from 0.001 to 100 ng/mL.

### Quartz Crystal Microbalance Immunosensors

A quartz crystal microbalance (QCM) is a quality testing instrument with a high sensitivity and has often been used as the conduction element in piezoelectric biosensors. The key technology for QCM immunosensors is to utilize the piezoelectric characteristics of quartz crystal resonators.

Tang et al. (2018) utilized a signal-on competitive QCM immunosensor for monitoring AFB1 in food (Figure 4B). In this method, a complex of AFB1–BSA and Con A was immobilized on the surface of an Au substrate modified with thiolated β-cyclodextrin. Anti-AFB1 antibody-marked nanoliposomes were combined with AFB1-functionalized QCM probes. When Triton X-100 was added, the encapsulated glucose molecules would be lysed and released from the nanoliposomes and would combine with Con A owing to the powerful affinity of glucose for Con A. Subsequently, anti-AFB1-labeled Con A dissociated from the QCM probe, leading to an alteration in the QCM frequency. In the presence of AFB1, the toxin and the immobilized AFB1–BSA on the probe competed for the anti-AFB1 antibody marked on the nanoliposome. The more AFB1 that was present, the more nanoliposomes that could detach from the QCM, thus causing an increase in the QCM frequency. With the optimal factors, the LOD of this immunosensor could be as low as 0.83 ng/kg, and the linear range was 1.0 ng/kg–10 mg/kg. In another example, Chauhan et al. (2015) introduced a novel electrochemical piezoelectric immunosensor functionalized with a SAM. The SAM of 4-aminothiophenol (4-ATP) was modified on an Au-coated quartz crystal (6 MHz). The AFB1 antibody (aAFB1) was immobilized on the surface of the quartz crystal by the amide linkage between aAFB1 and 4-ATP. The change in the QCM frequency indicated the mass of AFB1. This immunosensor exhibited a linear range of 0.1–4.0 ng/mL. In addition, this immunoelectrode could be reused up to five or six times.

## Biosensors Based on Mips

An MIP is a synthetic polymer with a specific recognition function for a specific target (Ahmad et al., 2019). The polymer is self-assembled by a template molecule and functional monomers via the polymerization of crosslinkers. When the template molecule is removed, there are holes with multiple active sites that match the spatial configuration of the template molecule in the polymer. In this situation, the polymer selectively identifies the template molecule and its analogs. Therefore, MIPs can be employed as recognition elements in biosensors based on MIPs. Conventional MIPs have many advantages, such as high specificity and sensitivity, ease of operation, and inexpensiveness. However, incomplete template elimination and a lower utilization of binding sites are undeniable limitations. Therefore, developing improved MIPs is attracting growing interest. The key to the success of an MIP sensor is whether the MIP is fixed on the converter effectively. At present, there are three common fixing methods: *in situ* polymerization, physical coating, and electropolymerization. In addition, the number of applications of MIP sensors in mycotoxin detection is limited, and only two kinds of MIP sensors are introduced in this section.

### Fluorescence Biosensors Based on MIPs

Fluorescence analyses have the advantages of being highly sensitive and selective and thus are broadly used in biological sensing systems. Chmangui et al. (2019) constructed a fluorescent probe for aflatoxin (AF) recognition based on MIP-QDsChmangui et al., 2019. MIPs were synthesized by applying methacrylic acid (MAA) as a unit and 5,7-dimethoxycoumarin (DMC) as an artificial template. Mn-doped ZnS QDs, template, and monomer were mixed together, forming a fluorescent MIP by the self-assembly method. Therefore, the MIPs were coated with Mn-doped ZnS QDs, which successfully transformed the signal of the target into a fluorescence signal. This biosensor showed a high sensitivity to AF, with an LOD of 0.016 mg/L.

In recent years, the research hotspots of biosensor designs have been focused on on-site detection methods and technology. Due to their advantages of easy operation and detection capability in the field, smartphone-based biosensors have been reported on many times in the literature. Biosensors combining novel materials have been well received because this method avoids tedious instrument operation. Sergeyeva et al. (2019) reported an MIP biosensor based on a smartphone for AFB1 detection. MIP membranes with binding sites were constructed by *in situ* polymerization with acrylamide (AA) and 2-(diethylamino)ethylmethacrylate (AMPSA) as functional monomers. Under UV irradiation, AFB1 binding with MIPs could emit fluorescence, and the AFB1 concentration was directly proportional to the fluorescence intensity. In addition, the fluorescence signal was recorded by obtaining photographs with a cell phone camera and was analyzed using image analysis software. Moreover, the LOD of this smartphone-based optical biomimetic sensor was 20 ng/mL.

### QCM Biosensors Based on MIPs

**FIGURE 5:**
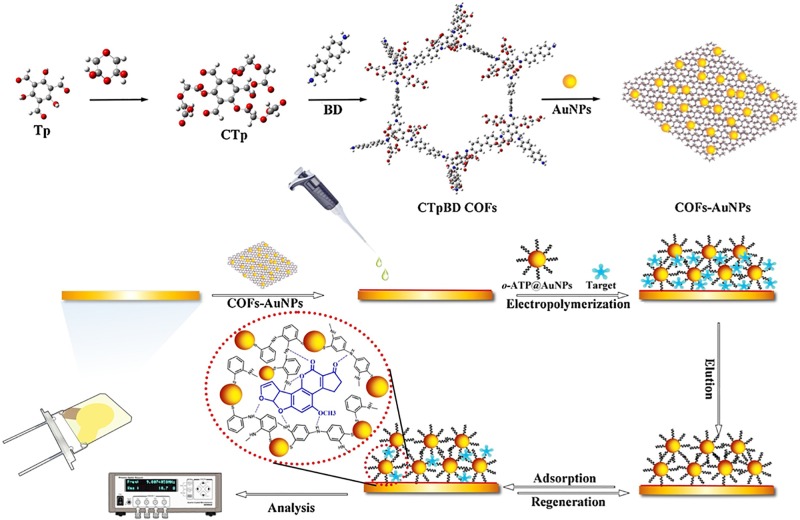
Schematic illustration for AFB1 determination of QCM-based MIP immonoassay. Modified from Gu et al. (2019).

QCM sensing systems consist of a quartz crystal and metal thin layer electrodes. The combined application of QCM and MIPs has received much attention in recent years (Baek et al., 2018; Battal et al., 2018; Gu et al., 2019; Zeilinger et al., 2019). Gu et al. (2019) developed a QCM-based biosensor for the determination of AFB1, which was fabricated by AuNPs by doping a molecularly imprinted layer on an AuNP-modified electrode (Figure 5). In this biosensor, an MIP membrane was synthesized by an electropolymerization method on the surface of the electrode. In addition, the MIP membrane synthesized in this way showed controllable film thickness and strong adhesion. The crosslink formed between the AuNPs and MIPs overcame the shortcomings of the MIPs because the AuNPs exhibited excellent electrochemical properties, favorable biocompatibility, and good chemical stabilization. Many recognition sites were established on the biosensor owing to the stereoscopic structure of the imprinted polymer and the large specific surface area of the AuNP base layer. When AFB1 was added, the mass changed, leading to a change in the crystal resonance frequency. Under optimal conditions, a low limit of detection of 2.8 pg/mL was achieved.

## Conclusion and Future Outlook

In the past decades, toxin contamination produced by biofilms has resulted in many negative effects. Moreover, mycotoxin contamination has become a serious challenge for preserving food and environmental safety and has received increasing attention worldwide. Therefore, diverse biosensors have been established for the detection of different low-concentration mycotoxins. In this review, the applications of biosensors for monitoring AFB1 from biofilms in the food field have been highlighted. Compared to other biosensors, optical biosensors possess a high selectivity for monitoring analytes with low concentrations. Moreover, electrochemical biosensors have received much attention because of their simplicity, ease of operation, and high selectivity.

In addition, we noticed a strong interest in the use of nanomaterials (noble metal nanoparticles, QDs, magnetic nanoparticles, and carbon-based nanoparticles) in biosensors due to the excellent optical, catalytic, and electrical properties of these nanomaterials. With the development of nanotechnology, novel nanomaterials, nanostructures, and the unique properties characteristic of these nanomaterials have been gradually discovered. Although the methods used to synthesize nanomaterials and the ability to control their sizes have attracted great interest, the above-mentioned factors remain a challenge. Moreover, the signal amplification strategy of biosensors is commonly used to detect analytes, specifically, low-concentration mycotoxins. Common signal amplifiers include hybridization chain reaction (HCR), the nuclease-assisted signal amplification strategy, AuNPs, the toehold-mediated DNA strand displacement reaction, and enzyme (e.g., HRP)-catalyzed amplification.

To date, the field of mycotoxin detection has achieved outstanding progress as more rapid, sensitive, and accurate methods have been developed. However, challenges and drawbacks remain in the application of biosensors for monitoring mycotoxins from biofilms. So far, researchers in related research fields seem to focus on constructing highly sensitive and selective biosensors, seeking simpler equipment and more rapid detection methods. However, researchers have overlooked an important detail: the reproducibility of biosensors. It is undeniable that large-scale instruments have great advantages in this respect. With the development of miniaturized portable instruments, the accuracy and reproducibility of biosensors are facing increased scrutiny. On the other hand, green detection methods and systems should be considered to avoid contributing to further contamination. For example, traditional metal QDs (CdS/CdTe/CuInS2 QDs) have a few drawbacks, such as strong toxicity of the metals to cells and difficult recovery. However, the above-mentioned issues can be avoided by using CDs as fluorescence probes, as CDs have the advantages of being non-toxic, environmentally friendly, widely available, and inexpensive. In addition, it is complicated and tedious to enrich low concentrations of mycotoxins in multicomponent food samples. This is a crucial step during the separation of AFB1 from small-molecule impurities in biofilms and could take a long time. In practical applications, the extraction process of mycotoxins from biofilms is still the greatest obstacle to achieving rapid on-site detection of mycotoxins. Developing multifunctional biosensors for simultaneous enrichment, separation, and detection will become an inevitable trend for on-site detection applications. Moreover, the degradation of AFB1 produced by biofilms after the end of the sample detection process is not a negligible task. Microbial fermentation and enzymolysis can reduce the toxicity of AFB1 in biofilms.

The combination of biosensors and nanomaterials will continue to expand with further development of this research field. Owing to the unique electrical, catalytic, and optical properties and other unknown properties of nanomaterials, biosensors based on nanomaterials will continue to be a research hotspot. Regarding the detection of mycotoxins in biofilms, on-site detection methods, especially dipstick test strip assays, have attracted the most attention. Biosensors based on dipstick test strips have many advantages, such as ease of use, user friendliness, inexpensiveness, and high sensitivity. Moreover, most biosensors based on dipstick test strips could be used to produce results observable by the naked eye, achieving qualitative measurements without large-scale instruments. Therefore, in these processes, there is much room for improving the sensitivity and accuracy from the lab to practical applications.

## Author Contributions

QW and WW drafted the manuscript. WW and QY designed the concept and revised the manuscript.

## Conflict of Interest

The authors declare that the research was conducted in the absence of any commercial or financial relationships that could be construed as a potential conflict of interest.
